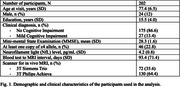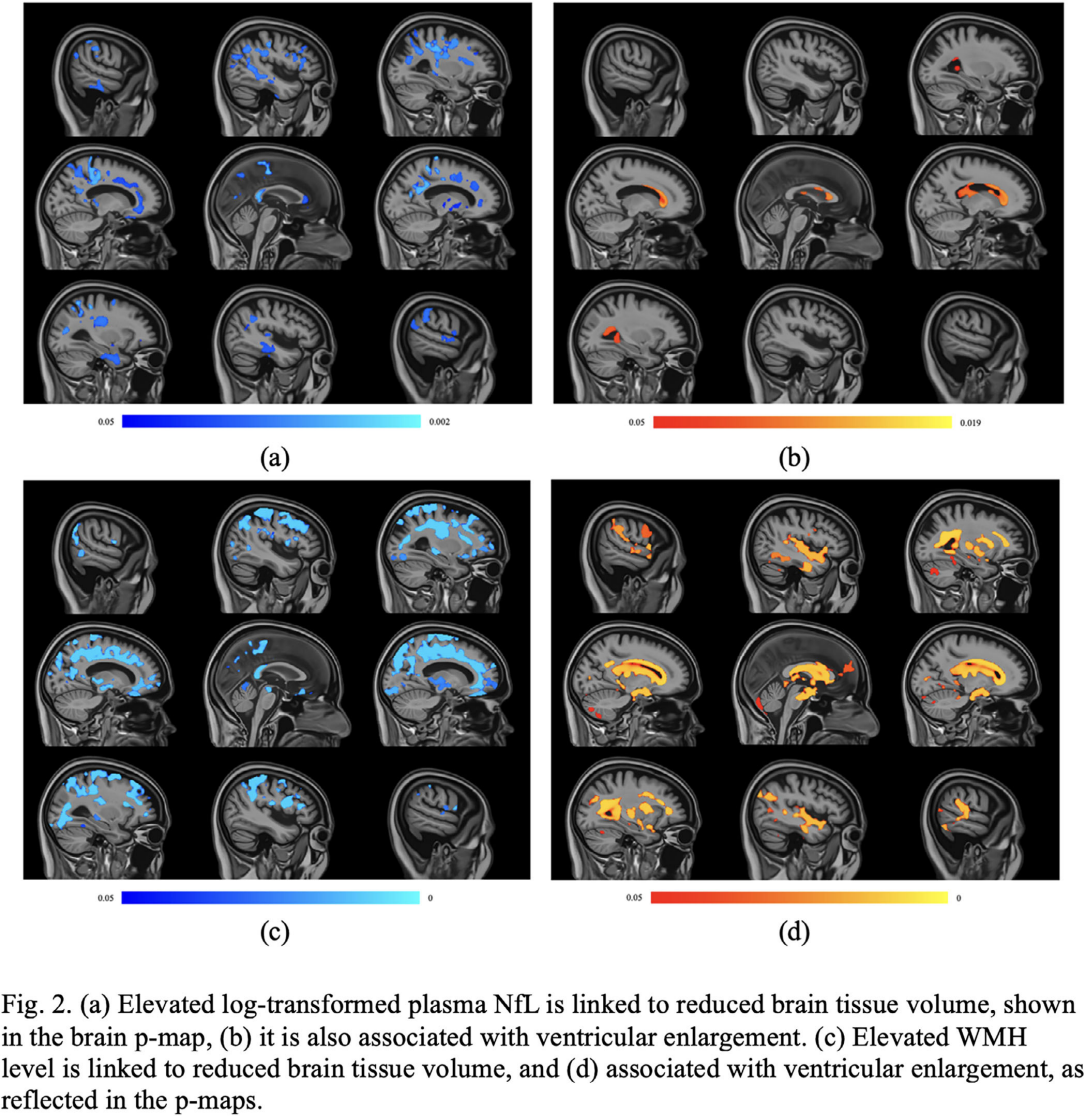# Plasma Neurofilament Light Links to Brain Tissue Loss, Ventricular Enlargement, and Elevated White Matter Hyperintensities

**DOI:** 10.1002/alz70862_110178

**Published:** 2025-12-23

**Authors:** Khalid Saifullah, Gulam Mahfuz Chowdhury, Arnold M Evia, David A. A. Bennett, Julie A Schneider, Konstantinos Arfanakis

**Affiliations:** ^1^ Illinois Institute of Technology, Chicago, IL USA; ^2^ Rush Alzheimer's Disease Center, Rush University Medical Center, Chicago, IL USA; ^3^ Rush Alzheimer's Disease Center, Chicago, IL USA

## Abstract

**Background:**

Neurofilament light chain (NfL), an axonal structural protein, has emerged as a promising blood biomarker for neurodegeneration and brain structural integrity. However, its relationship with white matter hyperintensities (WMH), a marker of small vessel disease, and direct neurodegeneration measured through deformation‐based morphometry (DBM) remains unclear in non‐demented older adults. This study aims to clarify the interplay between NfL, WMH, and neurodegeneration, with a specific focus on the role of vascular pathology as a mediator.

**Method:**

Participants and Data This study included 202 non‐demented community‐dwelling older adults from four studies: the Rush Memory and Aging Project, Religious Orders Study, Minority Aging Research Study, and African American Clinical Core (Figure 1). Fasting blood samples were processed and analyzed for NfL by NCRAD. Whole‐brain 3D T1w MPRAGE images were registered to the MIITRA atlas to calculate log Jacobian (LogJ) maps. WMH volume, segmented from T2w FLAIR and T1w images, was expressed as a percentage of intracranial volume and log‐transformed.

Statistical analysis Voxel‐wise linear regression was used to evaluate associations between NfL levels and LogJ maps, indicative of neurodegeneration, as well as between WMH burden and LogJ maps. Additionally, the relationship between NfL levels and WMH burden was analyzed. Finally we tested whether WMH mediates the relationship between NfL and neurodegeneration. All analyses adjusted for age, sex, education, time interval between blood sampling and MRI, and scanner type. Voxel‐wise analyses were conducted in FSL PALM with 5000 permutations, family‐wise error correction (*p* <0.05), and threshold‐free cluster enhancement.

**Result:**

NfL levels were significantly associated with neurodegeneration, evidenced by reduced brain tissue volume in key regions (Figure 2a) and with ventricular enlargement (Figure 2b). A positive association was also observed between NfL levels and WMH level (β = 0.0798, SE = 0.0436, *p* = 0.0344), even after adjusting for covariates. WMH burden itself was strongly associated with neurodegeneration (Figure 2c and 2d). Notably, the association between NfL and neurodegeneration was no longer significant after adjusting for WMH, underscoring WMH as a critical mediator.

**Conclusion:**

These findings highlight NfL as a biomarker reflecting neurodegenerative and vascular aspects of brain aging, with vascular pathology driving neurodegeneration.